# ScanGuard-YOLO: Enhancing X-ray Prohibited Item Detection with Significant Performance Gains

**DOI:** 10.3390/s24010102

**Published:** 2023-12-24

**Authors:** Xianning Huang, Yaping Zhang

**Affiliations:** School of Information Science and Technology, Yunnan Normal University, Kunming 650500, China; huangxn996@163.com

**Keywords:** X-ray image, prohibited items detection, deep learning, YOLOv5, multiscale feature fusion

## Abstract

To address the problem of low recall rate in the detection of prohibited items in X-ray images due to the severe object occlusion and complex background, an X-ray prohibited item detection network, ScanGuard-YOLO, based on the YOLOv5 architecture, is proposed to effectively improve the model’s recall rate and the comprehensive metric F1 score. Firstly, the RFB-s module was added to the end part of the backbone, and dilated convolution was used to increase the receptive field of the backbone network to better capture global features. In the neck section, the efficient RepGFPN module was employed to fuse multiscale information from the backbone output. This aimed to capture details and contextual information at various scales, thereby enhancing the model’s understanding and representation capability of the object. Secondly, a novel detection head was introduced to unify scale-awareness, spatial-awareness, and task-awareness altogether, which significantly improved the representation ability of the object detection heads. Finally, the bounding box regression loss function was defined as the WIOUv3 loss, effectively balancing the contribution of low-quality and high-quality samples to the loss. ScanGuard-YOLO was tested on OPIXray and HiXray datasets, showing significant improvements compared to the baseline model. The mean average precision (mAP@0.5) increased by 2.3% and 1.6%, the recall rate improved by 4.5% and 2%, and the F1 score increased by 2.3% and 1%, respectively. The experimental results demonstrate that ScanGuard-YOLO effectively enhances the detection capability of prohibited items in complex backgrounds and exhibits broad prospects for application.

## 1. Introduction

X-ray prohibited item detection holds significant value in the field of security, playing a crucial role in areas such as airports, railways, and border checkpoints. Traditional X-ray prohibited item detection methods often rely on manual operations or traditional algorithms, leading to low accuracy, low efficiency, and high dependence on human resources. Therefore, utilizing machine learning for automated prohibited item detection has become a research focus, offering wide-ranging application prospects.

In recent years, with the improvement of hardware computing power and graphics memory, deep convolutional neural networks have driven the development of computer vision fields such as object detection, instance segmentation, and keypoint detection, as well as a series of advances in X-ray prohibited item detection. Mu et al. [1] proposed a dilated dense convolution module for increasing the receptive field and feature expression ability by combining dilated convolution [2] and dense connection [3] for the problem of overlapping and obscuring each other’s texture information in the X-ray colour image, and introduced an attention mechanism to enhance the effective feature response and inhibit the influence of ineffective features to reduce the false negative rate of the model. Aiming at the problem of difficult detection of small and medium- sized items in baggage, Wu et al. [4] introduced the CBAM [5] attention mechanism into the YOLOX [6] backbone network to enhance the perception of prohibited items and added a bottom-up positive pyramid structure at the end of the neck portion for fusing more features of different sizes to increase the ability to detect small objects. Wang et al. [7] used ResNet-101 [8] as a backbone feature extraction network and fused the high-level feature maps with the low-level feature maps through skip connections, proposing a detection network that fused multiscale features, which effectively improved the detection and localisation accuracy of small objects. In order to balance the accuracy and speed of real-time detection of prohibited items in X-ray images, Li et al. [9] extended the YOLOv7 [10] backbone network with a MobileNetV3 block at the end, which captured global information while maintaining the lightweight architecture. Additionally, they integrated the CA [11] attention mechanism into the MPConv to enhance the model’s localization performance. Finally, they added a detection head with inputs of low-level and high-resolution feature maps to improve the model’s ability to detect small objects. Xiang et al. [12] proposed an enhanced network architecture to address the issue of severe object occlusion in X-ray prohibited item detection. This enhanced structure comprised multiscale smoothed atrous convolutions and a material-aware coordinate attention module. Through statistical analysis, it was found that the distribution of long-short sides in the target boxes was not equal for each type of prohibited item in different datasets. Therefore, they improved the SIOU [13] loss function and designed it as a long-short side decoupling module and a category information embedding module, which effectively mitigated the effect of different scale items on the detection accuracy. To address the low detection accuracy caused by intentionally hiding certain prohibited items, Wang et al. [14] created a large-scale dataset with high-quality bounding boxes and mask annotations. The dataset was divided into three subsets: easy, hard, and hidden, making it the first benchmark specifically designed for intentionally hiding prohibited items among clutter. The authors proposed a selective dense attention network (SDANet) to enhance the capturing ability of spatial and channel features. They also leveraged the dependencies between multiscale features to further improve the model’s performance.

Although existing detection algorithms have made some progress and can improve the accuracy and robustness of prohibited item detection to some extent, the algorithms’ recall and F1 score are generally low, mainly due to occlusion and object scale diversity. In practical applications, low recall means the model may miss certain prohibited items during detection, increasing security risks. To address this issue, this study investigated critical components within existing state-of-the-art models based on the YOLOv5 framework [15] and proposed a network model called ScanGuard-YOLO, tailored for the detection of prohibited items in X-ray security images. This model enhances feature representation via introducing the RFB-s [16] module at the end of the backbone network, employing dilated convolutions with varying dilation rates to expand the network’s receptive field. In the neck section, the efficient RepGFPN [17] module was employed to fuse multiscale features, and it utilizes a multi-layer aggregation connection mechanism and a reparameterization technique to further enhance fusion and detection performance. A dynamic head [18] was introduced to unify scale-aware, spatial-aware, and task-aware processing on the multiscale fused feature maps, enhancing the detection head’s ability to perceive objects of varying sizes and to handle occlusions. To further optimise the contribution of high-quality and low-quality samples to the regression loss, the bounding box regression loss function was defined as WIOUv3 [19]. The model proposed in this study was evaluated on two benchmark datasets, achieving significantly higher recall and F1 score than most current mainstream detection models. Compared to the state-of-the-art model YOLOv8s [20] with similar complexity, ScanGuard-YOLO improved recall and F1 score by 4.2% and 2.4%, respectively, on the OPIXray [21] dataset. On the HiXray [22] dataset, the recall and F1 values were comparable to that of YOLOv8s, but the precision of ScanGuard-YOLO was 1.1% higher than that of YOLOv8s. The experimental results show that ScanGuard-YOLO can effectively improve the performance of prohibited item detection in complex backgrounds, and has a wide range of practicality and great application potential in the field of automated security screening.

## 2. X-ray Security Image Prohibited Item Detection Model

In this section, we will introduce the relevant components of the YOLOv5 benchmark model and details of ScanGuard-YOLO. After that, we will introduce the RFB-s module, the efficient RepGFPN module, the dynamic head module, and the WIOUv3 loss.

### 2.1. YOLOv5 Baseline Model

YOLOv5, a one-stage object detection network introduced by the Ultralytics organisation, offers five basic network models, namely -n, -s, -m, -l, and -x, designed to meet different application scenarios and requirements. These models differ in the number of channels and modules and progressively increase computational and parameter complexity. In this study, we balanced between detection accuracy and speed, and selected YOLOv5s as the improved baseline model.

YOLOv5 comprises the feature extraction backbone, the neck feature pyramid fusion structure, and the detection head. The backbone section consists of four convolutional layers, four C3 modules, and an SPPF module, all designed for multiscale feature extraction. Among these, the C3 modules and the SPPF module serve as the core of the backbone. The neck section consists of a top-down feature pyramid network (FPN) [23] and a bottom-up path aggregation network (PAN) [24], enabling the fusion of shallow-level visual features and deep-level semantic features across different scales. The head part consists of a coupled 1 × 1 convolution for predicting different scale objects on the output feature maps of three different sizes, where the deeper feature map has a larger receptive field for detecting larger objects.

### 2.2. Our Method

**Figure 1:**
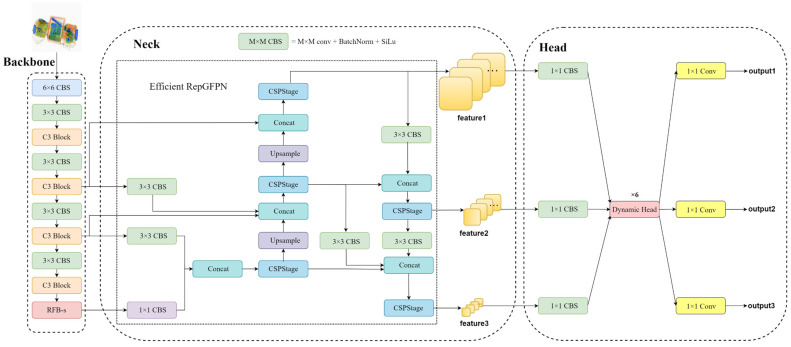
ScanGuard-YOLO architecture diagram.

Different objects with various shapes, densities, and thicknesses in X-ray imaging exhibit different degrees of scattering and absorption. These effects can impact the quality and clarity of the resulting images. Moreover, multiple objects may overlap in the image, making it difficult to distinguish their boundaries. This complexity poses challenges for detection tasks, especially when smaller prohibited items are obscured by larger objects, leading to missed detections and lower recall rates for the model. The original YOLOv5 model struggles to effectively address these issues. To tackle these challenges, we propose ScanGuard-YOLO, whose overall network architecture is depicted in Figure 1. Specific input/output details are given in Table 1. The SPPF module within the baseline model’s backbone is a spatial pyramid fusion module that samples the input feature map using pooling kernels of different sizes. It subsequently concatenates multiple sampled feature maps in the depth dimension to create a feature representation with rich semantic information. However, this module employs max-pooling for sampling, which discards some pixels in the input feature map, resulting in the loss of certain fine-grained details. The max-pooling operation requires comparing the pixels within each pooling window and selecting the largest as the output feature, which is computationally intensive. Hence, in this study, we replaced the SPPF module with the RFB-s module, which expands the receptive field without reducing resolution, enhancing the network’s ability to perceive global information. In the neck section, an efficient multiscale feature fusion module, termed efficient RepGFPN, was constructed. Its purpose is to enhance interactions between features of varying scales, allowing for better adaptation to changes in object scale across different scenarios. To cope with the data distribution characteristics of different X-ray imaged prohibited item datasets, a detection head containing an attention mechanism was introduced to enhance the correlation after fusing the features and to help the model focus on useful features. Finally, the bounding box regression loss was designed as a WIOUv3 loss to optimise the training strategy of the model for hard and easy samples to improve the accuracy of the model.

#### 2.2.1. Expanding the Receptive Field

**Figure 2:**
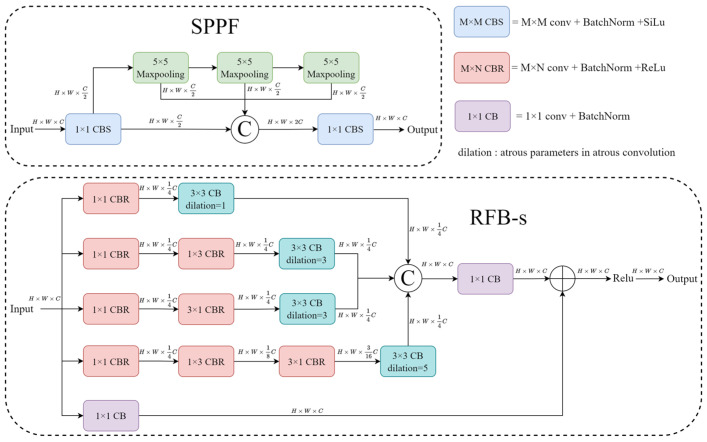
Structure of SPPF and RFB-s.

When performing feature extraction, it is common to use max-pooling operations with varying kernel sizes to extract features for objects of different sizes within the feature maps. However, max-pooling reduces the size of feature maps by selecting the maximum value within specific regions, which can result in the loss of certain feature information. In X-ray prohibited item images, there are often multiple items obscuring each other, which causes us to observe only some of the edge features. In order to better capture the features and structures in the input data to enhance the perception of objects of different sizes, we adopted the strategy of expanding the receptive field in the feature extraction network. Specifically, the RFB-s module was introduced, and its structure is shown in Figure 2.

This module utilizes multiple dilated convolutions with different dilation rates to enlarge the neural network’s receptive field. Concurrently, it incorporates multiple convolutional kernels of varying dimensional sizes to learn abstract features at different levels, thereby enhancing the model’s expressive capacity and better representing object shape, texture, and other information. Furthermore, the substitution of 3 × 3 convolutions with 1 × 3 and 3 × 1 convolutions was employed to reduce model parameters and alleviate computational overhead. This design contributes to efficient feature extraction, especially in situations with limited computational resources.

#### 2.2.2. Efficient Multiscale Feature Fusion

**Figure 3:**
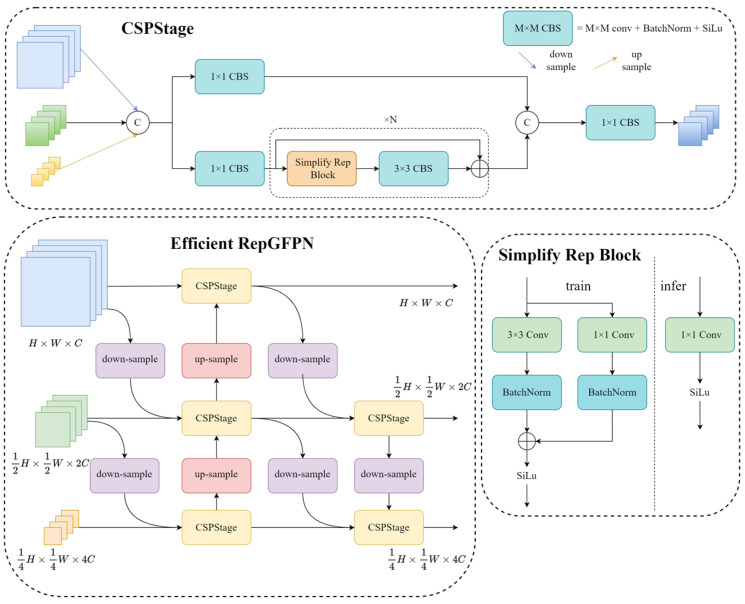
Structure of the efficient RepGFPN.

The backbone network is usually a deep convolutional neural network for extracting feature representations of an image from shallow to deep layers, with different receptive fields and semantic information at different levels. The traditional feature pyramid proposes a top-down feature fusion strategy, in which the upsampled deep feature maps are sequentially fused with the shallow feature maps through a top-down path to obtain a feature pyramid with multiscale information. However, using only one top-down unidirectional information flow path, the deeper feature maps may have lost some detailed information [23]. To compensate for the lack of unidirectional data flow, PANet [24] adds a bottom-up feature propagation mechanism. PANet utilizes feature propagation modules to propagate feature information between different levels, enhancing the interaction between features at different levels. In order to improve the detection ability of different scale objects, we constructed an efficient multiscale feature fusion module, efficient RepGFPN, at the neck of the network, the structure of which is shown in Figure 3.

In this structure, when fusing multiscale features, we employed a non-shared channel setting, which allowed for the preservation of the original feature information at each scale. Since the number of channels is not adjusted via convolution, the downscaling and upscaling operations of the features are avoided, which reduces the loss of information. At the same time, a richer feature representation is formed via the stacking features from different scales according to the original channels, which helps the network better understand and use the multiscale information. In the Simplify Rep Block structure, a structural re-parameterization mechanism is employed to eliminate branches that are present during training but not required during inference. This helps reduce the model’s computational requirements and memory footprint, thereby improving the model’s inference efficiency.

#### 2.2.3. Dynamic Detection Head

**Figure 4:**
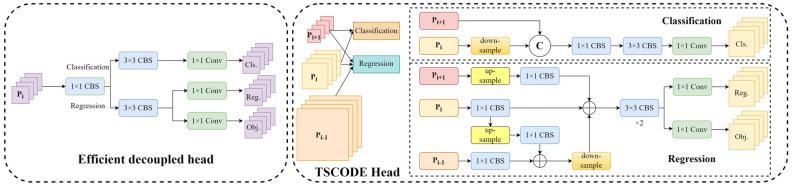
Structure of efficient decoupled head and TSCODE head.

In YOLOv5, 1 × 1 convolutions are used to perform classification and regression on the three scale features output by the neck. In the YOLOX model, the detection head’s classification and regression operations are decoupled, accelerating model convergence. To reduce model computational complexity, YOLOv6 [25] employs a hybrid-channel strategy and reduces two 3 × 3 convolutions in the YOLOX detection head to one, achieving lower inference latency, referred to as the efficient decoupled head. All the above detection heads are only applied to feature maps of the same scale without considering multiscale contextual features. Zhuang et al. [26] proposed a context-decoupled head that fused multiscale features, called the TSCODE head, whose structure is shown in Figure 4. The detection performance of the model was further improved by fusing small-scale features with high-level semantic information for the classification task while using features from all scales for regression. However, the above detection heads do not consider unity, adaptability, and multi-task support. Furthermore, the performance of the decoupled head may not necessarily be superior to that of the coupled head after improvements to multiple modules of the network. Adjustments should be made based on actual application scenarios, and specific ablation experiments are described in Section 3.4.2.

**Figure 5:**
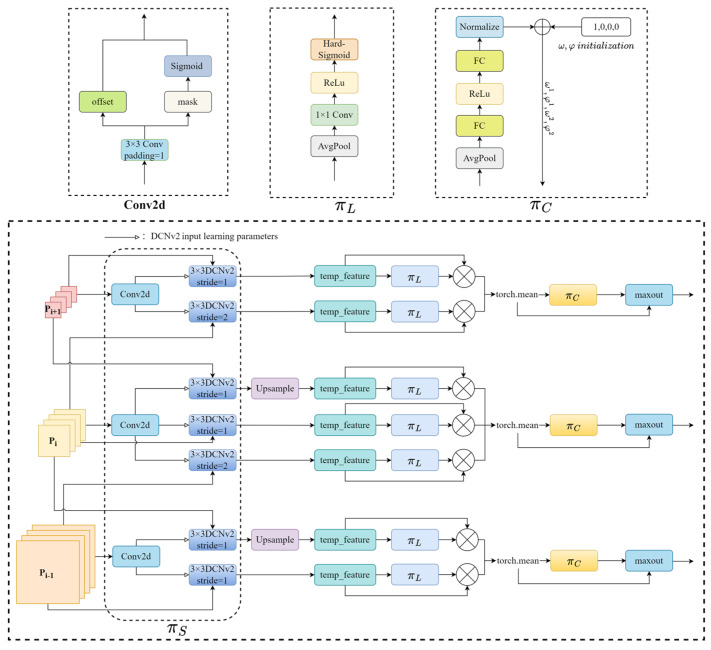
Structure of the dynamic head.

In X-ray prohibited item detection tasks, there can be various categories of prohibited items within packages, and these items come in different sizes and levels of occlusion. Therefore, it is essential for the detection head to possess scale-awareness capability. Secondly, the prohibited items to be detected may present different shapes and appear in any position in the image under different viewpoints, thus requiring spatial-awareness of the detection head. Finally, due to the varying contributions of different channel features to different tasks (e.g., classification and regression), it is necessary to dynamically adjust the detection head’s attention allocation to each channel based on the task type. Hence, there is a need to enhance the task-awareness capability of the detection head. For this reason, a dynamic detection head [18] was constructed in the head, and its structure is shown in Figure 5. The effective fusion between scale-awareness, spatial-awareness and task-awareness was achieved by introducing the attention mechanism, and the attention formula is expressed as:

(1) $$W\left(F\right)=\pi \left(F\right)\cdot F$$

where, given an input tensor $F\in R^{L\times S\times C}$, *L* was the number of different scale feature maps outputted through the neck section, *S* was the product of the width and height of the feature maps, *C* was the number of channels of the feature maps, and $\pi \left(\cdot \right)$ was the attention function.

To simplify the formula, we still denote the output after the attention operation as *F*. Thus, the formula representing the concatenation of spatial-awareness $\pi_{S}\left(\cdot \right)$, scale-awareness $\pi_{L}\left(\cdot \right)$, and task-awareness $\pi_{C}\left(\cdot \right)$ attention mechanisms can be expressed as:

(2) $$W\left(F\right)=\pi_{C}(\pi_{L}(\pi_{S}\left(F\right)\cdot F)\cdot F)\cdot F$$

In the prohibited items detection task, objects can be deformed due to different viewing angles, and traditional fixed-shape convolution kernels and fixed-size receptive fields often struggle to adapt well to this situation. Therefore, we require a convolution operation that can adaptively adjust the receptive field and convolution kernel sampling positions to better capture the deformation information of the objects. The spatial-aware attention mechanism uses deformable ConvNets v2 (DCNv2) [27] to adjust the size of the convolutional kernel receptive field. DCNv2 can not only adjust the offset of input features but also modulate the sampling point weights. By learning the offsets, it becomes possible to adaptively adjust the sampling positions of the convolutional kernel on the input feature map. This enables the network to better accommodate variations such as deformations and local changes in the presence of prohibited items at different locations. By learning the weights of sampling points, the network can adjust the sampling weights of convolutional kernels at different positions more effectively based on different local structures and their feature importance. The formula for spatial-aware attention is expressed as:

(3) $$\pi_{S}\left(F\right)\cdot F=\frac{1}{L}\overset{L}{\underset{l=1}{\sum }}\overset{K}{\underset{k=1}{\sum }}w_{l,k}\cdot F\left(l;p_{k}+\Delta p_{k};c\right)\cdot \Delta m_{k}$$

Here, $F\in R^{L\times S\times C}$ was the input tensor, and *c* denoted *c*-th channel of the feature map. K represented the number of sampled pixels in the convolution kernel, $p_{k}$ was the *k*-th sampled pixel in the convolution kernel, $w_{k}$ corresponded to its weight, $\Delta p_{k}$ denoted the learned offset, and $\Delta m_{k}$ was an importance scalar obtained via learning at $p_{k}$.

Scale-aware attention performs adaptive average pooling, a 1 × 1 convolution, and a hard-sigmoid activation on the *S* and *C* dimensions of the features, which generates attention weights specific to different scale features. These weights dynamically fuse features of different scales based on their semantic importance, as shown in Figure 5, which can effectively improve the performance of the model on the multiscale detection task. The scale-aware attention formula is expressed as:

(4) $$\pi_{L}\left(F\right)\cdot F=\sigma \left(f\left(\frac{1}{SC}\underset{S,C}{\sum }F\right)\right)\cdot F$$

(5) $$\sigma \left(x\right)=max\left(0,min\left(1,\frac{x+1}{2}\right)\right)$$

In the equation, $f\left(\cdot \right)$ represented a linear function approximated by a 1 × 1 convolution, and $\sigma \left(x\right)$ denoted the hard-sigmoid activation function.

The task-aware attention was inspired by the dynamic ReLU [28], which adaptively learns the importance of each feature channel and reallocates the weights of individual channels based on different tasks. The task-aware attention formula is expressed as:

(6) $$\pi_{C}\left(F\right)\cdot F=max\left(\omega^{1}\left(F\right)\cdot F_{c}+\varphi^{1}\left(F\right),\omega^{2}\left(F\right)\cdot F_{c}+\varphi^{2}\left(F\right)\right)$$

Here, $F_{c\;}$ represented the *c*-th channel of the feature map, and ${\left[\omega^{1},\omega^{2},\varphi^{1},\varphi^{2}\right]}^{T}=\theta \left(\cdot \right)$ denoted the hyperfunction for learning control activation thresholds. First, global average pooling was applied on the *S* dimensions of the feature map to compute the mean value of each channel, resulting in a fixed-size vector. The compressed feature vector was further processed through two fully connected layers, whose outputs indicated the relative importance weights of each channel in different tasks. To ensure that the task-aware attention weights have positive and negative values, a shifted sigmoid function was used to normalise the output within the range of [−1, 1]. Subsequently, these values were passed through the hyperfunction $\theta \left(\cdot \right)$ to generate four learnable parameters,$\;\omega^{1},\omega^{2},\varphi^{1},\varphi^{2}$. These parameters were used in subsequent maxout operations to activate different channels of the input feature map. In this way, the obtained attention weights can enhance the features of specific channels and suppress the features of other channels.

#### 2.2.4. Bounding Box Regression Loss Function

The loss function is a metric that measures the discrepancy between model predictions and ground truth. It is used to evaluate the model’s performance and guide parameter updates. The loss function of YOLOv5 consists of classification loss, confidence loss, and bounding box regression loss. In order to improve the precision and recall of detection and to accelerate the speed of bounding box regression, we introduced WIOUv3 [19] as the bounding box regression loss. WIOUv3 adopts a dynamic and non-monotonic focusing mechanism, compared to Focal-EIOU’s [29] static focusing mechanism, which can more effectively balance the contribution of high-quality and low-quality samples to the loss function. Its formula is represented as follows:

(7) $$L_{WIOUv3}=rL_{IOU}e^{\left(\frac{{\left(x-x_{gt}\right)}^{2}+{\left(y-y_{gt}\right)}^{2}}{{\left(w_{c}^{2}+h_{c}^{2}\right)}^{*}}\right)}$$

(8) $$r=\frac{\beta }{\delta \vartheta^{\beta -\delta }}$$

(9) $$\beta =\frac{L_{IOU}^{*}}{\bar{L_{IOU}}}$$

(10) $$L_{IOU}=1-IOU$$

where $x_{gt}$ and $y_{gt\;}$ denoted the coordinates of the centre of the ground truth bounding box, and x and y represented the coordinates of the center of the predicted bounding box. Furthermore, $w_{c}$ and $h_{c}$ stood for the width and height of the minimum enclosing rectangle of the predicted and ground truth boxes. $L_{IOU}^{*}$ indicated that during the computation of the loss within the current batch, $L_{IOU}$ was detached from the computation graph, making it devoid of gradient information. $\bar{L_{IOU}}$ was the exponential running average with momentum factor $m=1-\sqrt[{7000}]{0.5}$, and β was the ratio between $L_{IOU}^{*}$ and $\bar{L_{IOU}}$, which characterized the outlier degree used to describe anchor box quality; a smaller outlier degree implies higher anchor box quality. r was the nonmonotonic focusing coefficient of β, with hyperparameters ϑ=1.9 and δ=3.0 during training. The WIOUv3 loss not only reduced the competitiveness of high-quality anchor boxes but also mitigated the harmful gradients generated by low-quality samples. This allows the network to focus more on anchor boxes of moderate quality, thereby enhancing the overall accuracy of object detection.

## 3. Experiments

This section introduces the dataset, experimental setup, and evaluation metrics used in our experiments. Next, we validated the state-of-the-art performance of the proposed model through comparative experiments. Finally, we assessed the effectiveness of our approach through ablation experiments.

### 3.1. Datasets

OPIXray [21] is a dataset for occluded prohibited items detection using X-ray imaging. It comprises 8885 images and includes five classes of sharp objects commonly carried by passengers: folding knives, straight knives, scissors, utility knives, and multi-tool knives.

HiXray [22] is a large, high-quality dataset for prohibited item detection in X-ray images. It consists of a total of 45,364 images, encompassing eight classes of items commonly carried by passengers, including two types of convenient portable chargers, water bottles, laptops, mobile phones, tablets, canned cosmetics, and nonmetallic lighters.

The OPIXray dataset is artificially synthesized via scanning through the security screening machine, while the HiXray dataset was collected from real daily security checks at international airports, and two datasets of prohibited items were manually labelled by professional security screeners at international airports. All images are stored in JPG format with an average resolution of 1200 × 900 and a maximum resolution of 2000 × 1040.

### 3.2. Experimental Environment and Evaluation Indicators

The following experiments were conducted on a server with an NVIDIA A40 (48 GB) GPU, using the PyTorch 1.13.1 framework for training and testing. Both datasets were divided into training, validation, and test sets in a ratio of 6:2:2. In all the experiments described below, the models were trained for 100 epochs, with an input image size of 1280 × 1280 and a batch size of 16. We used the SGD optimizer with an initial learning rate of $\;10^{-2}$, a final learning rate of $\;10^{-4}$, momentum of 0.937, weight decay of $5\times 10^{-4}$, and pretrained weights were used.

We employed several metrics to evaluate the model’s prediction performance, including the mean average precision (mAP@0.5, with an IOU threshold greater than 0.5), precision, recall, and F1 score.

### 3.3. Comparative Experiments with State-of-the-Art Models

We compared the proposed ScanGuard-YOLO model with several excellent object detection models, including YOLOv5, YOLOv6, YOLOv7, and YOLOv8, on the OPIXray and HiXray datasets to validate the superiority of ScanGuard-YOLO over similar models. In all tables below, bold indicates the best-performing values, and underlined values represent the second-best results. The baseline model was YOLOv5s 7.0.

The results of the comparison experiments between different models on the OPIXray dataset are shown in Table 2. It can be seen that compared to the baseline model, ScanGuard-YOLO improved recall by 4.5%, mAP(@0.5) by 2.3%, F1 score by 2.3%, and precision was slightly reduced by 0.1%. Despite the increase in computation and number of parameters, ScanGuard-YOLO still shows the best overall performance compared to state-of-the-art models with more parameters.

The results of the comparison experiments between different models on the HiXray dataset are shown in Table 3. It was found that mAP(@0.5) was 1.6% higher than the baseline model, which was the optimal value among several comparison models. Although the recall and F1 score were only sub-optimal, they were only 0.2% and 0.3% lower than the optimal values. Considering all evaluation metrics and the number of parameters and calculations, ScanGuard-YOLO was more suitable for X-ray prohibited item detection tasks.

On the OPIXray dataset, which had a smaller volume of data, ScanGuard-YOLO surpassed the models in Table 2. The model’s noticeable performance boost can be credited to proficiently using the limited dataset, which facilitated capturing relevant features and generalized well in this constrained surrounding. In contrast, the challenges faced while working with the HiXray dataset, with a larger data volume and elevated category diversity, adversely affected the model’s performance. While our model may not surpass the performance enhancement achieved on OPIXray, it is important to note that it consistently achieved optimal results in terms of the primary evaluation metric, mAP(@0.5), as shown in Table 3. The prioritisation of mAP(@0.5) ensured that the model excelled in achieving the necessary precision for detecting prohibited items in X-ray imaging, aligning with real-world security applications’ stringent requirements.

YOLOv7 showed suboptimal performance on the smaller OPIXray dataset. However, on the larger HiXray dataset, YOLOv7 showed excellent performance. This could be attributed to the fact that YOLOv7 had a larger number of parameters, potentially leading to overfitting on the smaller OPIXray dataset. Therefore, ScanGuard-YOLO showed superior performance than YOLOv7 on the OPIXray dataset.

To verify whether our approach performed exceptionally well with a comparable number of parameters to YOLOv7, we built the ScanGuard-YOLOm model by extending the YOLOv5m model with the addition of the RFB-s, efficient RepGFPN, dynamic head modules, and the WIOUv3 loss. As shown in Table 3, ScanGuard-YOLOm outperformed YOLOv7 in all metrics, indicating that with the same number of parameters, ScanGuard-YOLOm may perform better in feature extraction and feature fusion, and can capture more useful information from the input data, resulting in better performance metrics.

**Figure 6:**
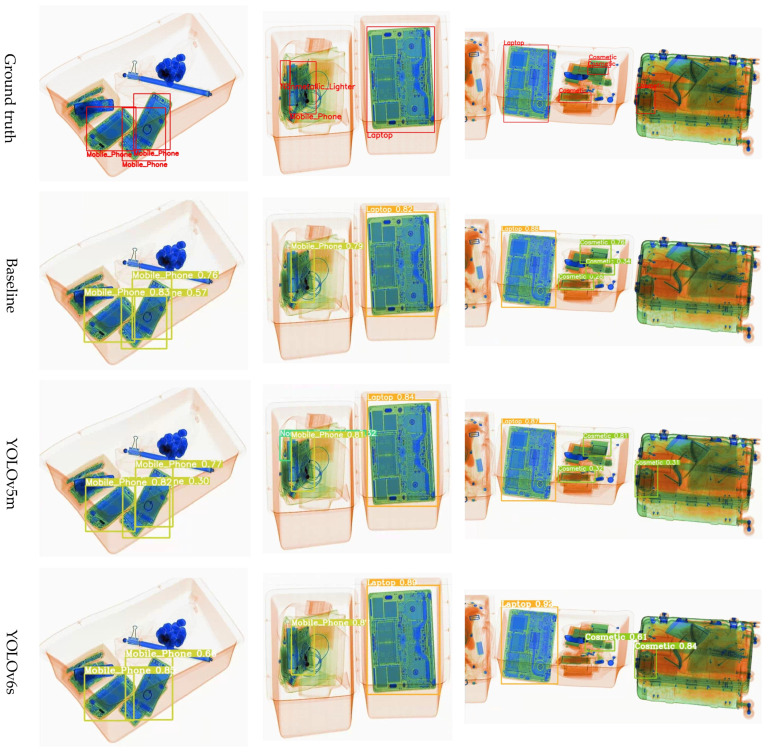

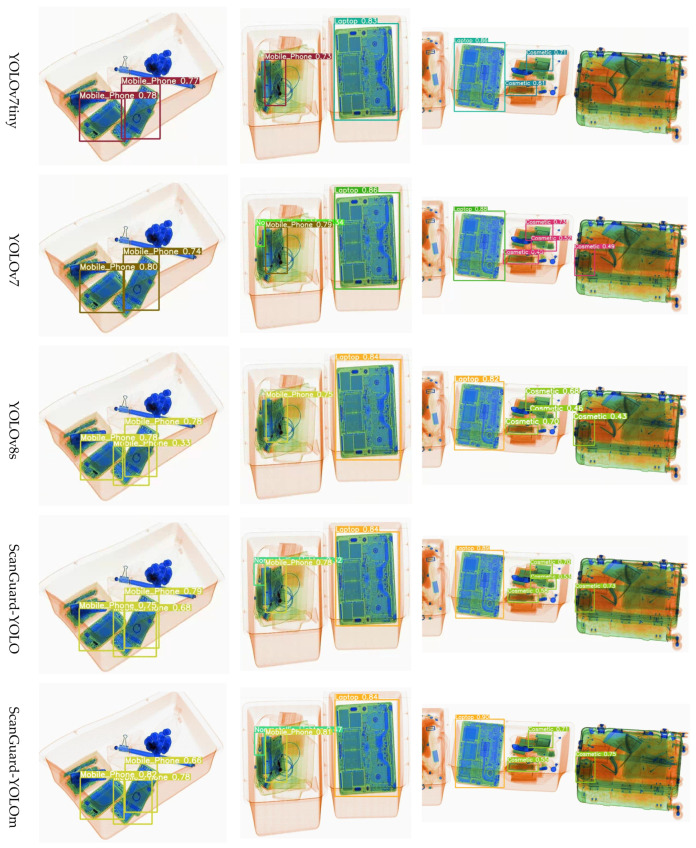
Comparison of detection performance of different detection models on the HiXray dataset.

Figure 6 illustrates the comparison of the detection performance of ScanGuard-YOLO with the aforementioned advanced models on the HiXray dataset. We found that ScanGuard-YOLO was more effective at detecting occluded objects with the same number of parameters, while other advanced models tended to have more instances of missed detections. Even when all models detected prohibited items, ScanGuard-YOLO captured more global and multiscale information, enhancing the perceptual capabilities of its dynamic head. This results in higher confidence scores for detected prohibited items.

### 3.4. Ablation Experiment

In this section, YOLOv5s 7.0 was used as the baseline model for ablation studies on the OPIXray dataset to demonstrate the effectiveness of the proposed model. The experimental strategies for each group of experiments were consistent. The symbol “✓” represents the addition of corresponding modules in all the experiments mentioned below.

#### 3.4.1. Impact of Different Modules on Model Performance

In Table 4, A is the efficient RepGFPN module, B is the WIOUv3 loss, C is the dynamic head module, and D is the RFB-s module. It can be observed that when the A module was added alone, mAP(@0.5) decreased by 0.2%, but the recall rate increased by 1.4%, indicating that more prohibited items were detected. Furthermore, through combination experiments of different modules, we observed that as the improved modules were gradually added to the network, the value of mAP(@0.5) also increased gradually. This indicated that there may be a synergistic effect between the improvement modules, leading to better performance. When all four modules were stacked together in the network, the model achieved the best performance, with a 4.5% increase in recall, a 2.3% increase in mAP(@0.5), and a 2.3% increase in F1 score. These improvements enabled the model to more accurately predict the categories of prohibited items to be detected, resulting in increased reliability and usability in practical applications.

Through ablation experiments with different modules, we observed that the RFB-s module had a significant impact on precision, while the efficient RepGFPN and dynamic head modules had a more pronounced impact on recall. When combined with the WIOUv3 loss function and the other three modules, optimal performance was achieved. In Section 3.4.2 and Section 3.4.4, we performed ablation studies on various excellent detection head modules and loss functions.

**Figure 7:**
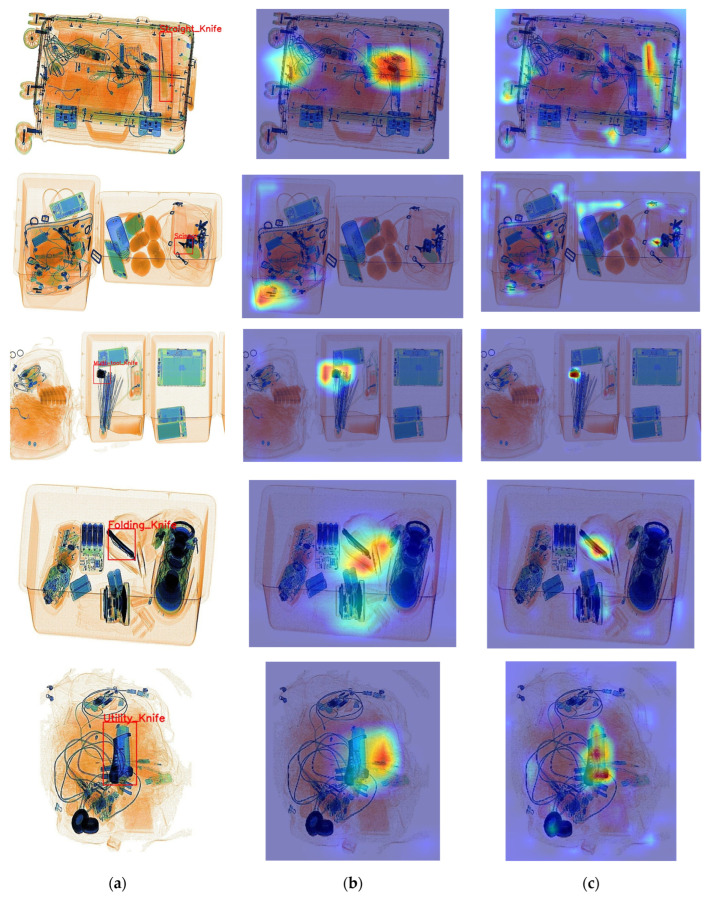
Comparison of heatmap effects between ScanGuard-YOLO and the baseline model. (**a**) Ground truth. (**b**) Heatmap of the baseline model. (**c**) Heatmap of ScanGuard-YOLO.

According to Table 4, it is clear that the baseline model tended to produce false negatives during detection, resulting in lower recall. In the context of prohibited item detection, minimizing false negatives is of paramount importance. To demonstrate the detection performance improvement achieved by ScanGuard-YOLO, we used the Grad-CAM++ [30] method for heatmap visualization, as shown in Figure 7. Heatmaps were used to visualise the regions of interest that the model focused on during the detection task. In these heatmaps, the red regions indicated higher contribution levels, while the blue regions represented lower contribution levels. The heatmap results show that ScanGuard-YOLO had stronger perceptual capabilities than the baseline model. It can more accurately detect features related to the prediction results, reducing the reliance on irrelevant features and resulting in a more accurate object box regression.

**Figure 8:**
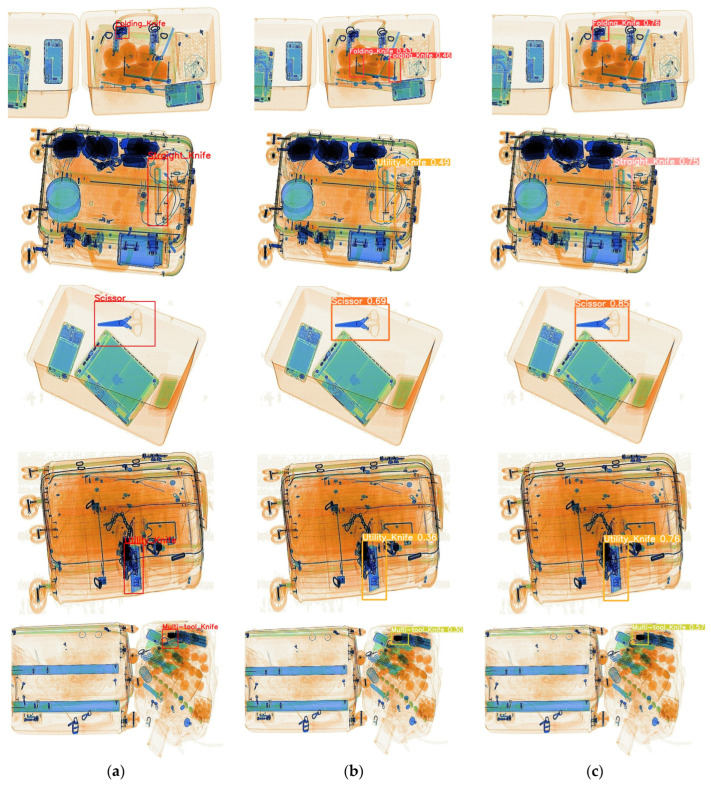
Comparison of actual detection performance between ScanGuard YOLO and the baseline model. (**a**) Ground truth. (**b**) Baseline model detection results. (**c**) ScanGuard-YOLO detection results.

Figure 8 compares the actual detection performance between ScanGuard-YOLO and the baseline model for five categories of prohibited items. In the “folding knife” category detection, occlusion of the object led to missed detections and false positives in the baseline model, while ScanGuard-YOLO was able to identify the category of occluded objects with higher confidence scores. In the case of the “straight knife” category detection, where there were many densely distributed objects, the baseline model experienced false positives. For the remaining three category detections, the baseline model achieved lower confidence scores, while ScanGuard-YOLO achieved higher scores.

As can be seen from the heat map and the images of the actual detection effect, ScanGuard-YOLO detected better than the baseline model in images with object occlusion and dense distribution of multiscale objects and achieved the expected effect.

#### 3.4.2. The Impact of Different Detection Heads on Model Performance

In this section, experiments were conducted on several excellent detection head methods to investigate the impact of different detection heads on the performance of both the baseline model and the proposed model.

Table 5 shows the comparative experimental results of four detection heads on the baseline model, where a represents the efficient decoupled head, b represents the TSCODE head, c represents the replacement of the 1 × 1 convolution at the end of the dynamic head with the efficient decoupled head, and d represents the dynamic head. If none of the options a, b, c, or d were used, it corresponds to the baseline model with a 1 × 1 convolution detection head. The experimental results indicated that the use of c on the baseline model achieved higher precision and recall compared to d. Due to the decoupled head, which handled the prediction of position and category separately, their learning objectives were more explicit and independent. This independence can help the network to better capture both the position and category information of the targets, resulting in higher precision and recall. However, the performance of the coupled option was also excellent, suggesting that task-awareness within the dynamic detection head played a crucial role.

Table 6 shows the comparative experimental results of adding different detection heads to the baseline model after incorporating the modules of RFB-s, efficient RepGFPN, and WIOUv3 loss. We observed that in the combined module experiment, the performance of the dynamic head after decoupling was not as good as that of the coupled head with fewer parameters. This may be due to the introduction of the RFB-s and efficient RepGFPN modules, which enhance the feature extraction and fusion capabilities of the model, resulting in more powerful feature representations after fusion. In this scenario, the task-aware module within the dynamic head can perform well without decoupling the coupled head and training separate regressors and classifiers. This suggests that a more comprehensive model-based improvement in the X-ray prohibited item detection task may lead to more significant performance gains beyond simply adding decoupled or coupled heads.

#### 3.4.3. The Impact of the Number of Input Channels of the Detection Head and Its Module Repetitions on Model Performance

To investigate the impact of the number of input channels on the performance of the dynamic head, we conducted relevant ablation experiments on the baseline model, as shown in Table 7 (with a module repetition number of 6). The highest mAP(@0.5) was achieved when the number of input channels was set to 512. This suggested that a larger number of input channels provided richer feature representations, which helped the network to learn more complex features and patterns. However, as the number of channels increased, the model became more complex and required more computational resources during inference. When the input channel number was adjusted to 128, the mAP(@0.5) reached its second-highest value, but the number of parameters was reduced by a factor of 5 compared to the input channel number of 512. While fewer input channels limited the model’s representational capacity, the reduced parameter and computational requirements made the model lighter, making it more suitable for real-time detection of prohibited items.

To further investigate the impact of the number of dynamic head modules on model performance, we set the number of input channels to 128 and performed ablation experiments on the baseline model, as shown in Table 8. The purpose of repetitive modules was typically to increase the feature extraction and representation capabilities of the model. Each repetitive module can learn different levels of abstract feature representations and combine these features to better understand the input data. However, too many modules can lead to an overly complex model that overfits the training data and does not generalise well to new data. In experiments, six dynamic head modules achieved the best performance on the OPIXray dataset.

#### 3.4.4. The Impact of Different Loss Functions on Model Performance

**Figure 9:**
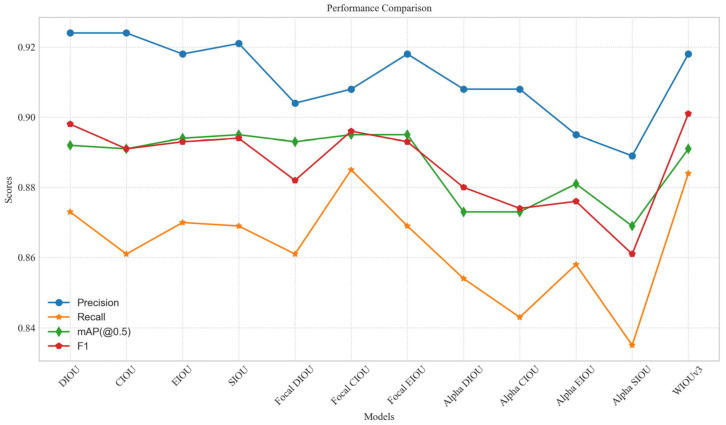
Performance comparison line chart for different loss functions.

In this section, experiments were conducted to explore the impact of various commonly used loss functions on both the baseline model and the proposed model. Table 9 and Figure 9 illustrate the effect of different loss functions on the performance of the baseline model. The baseline model used the CIOU loss for bounding box regression and achieved the highest precision but relatively low recall. X-ray prohibited item detection required high recall, as low recall implied a significant number of false negatives, which could lead to serious security problems. Compared to the other loss functions, the WIOUv3 loss had the highest F1 score, with a recall that was only 0.001 lower than the highest recall.

**Figure 10:**
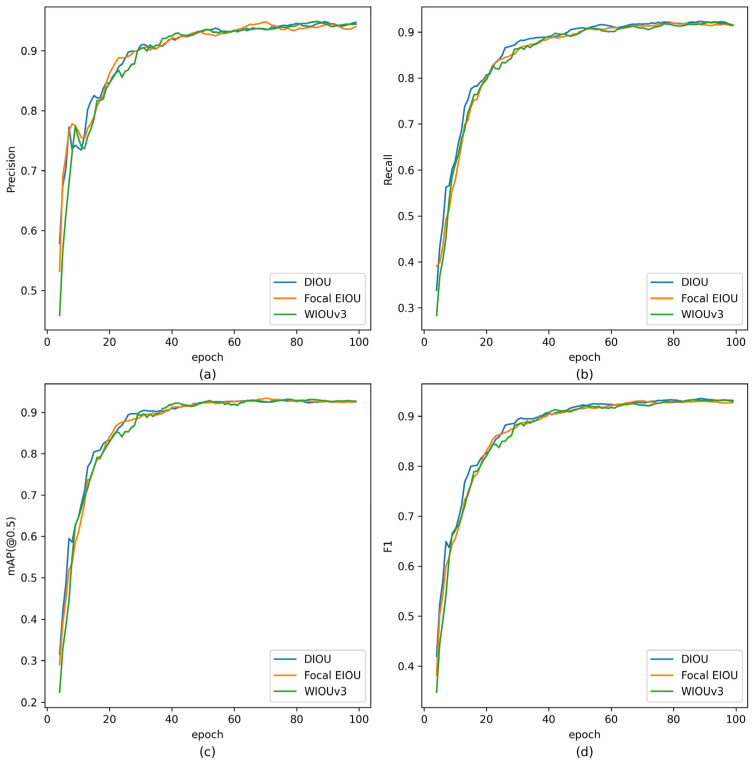
Performance plot on the validation set for each epoch during training. (**a**) Precision, (**b**) Recall, (**c**) mAP(@0.5), (**d**) F1.

To investigate whether the previously superior performance of the loss functions holds when combined with the RFB-s, efficient RepGFPN, and dynamic head modules on the baseline model, we conducted further ablation experiments, and the results are shown in Table 10. The WIOUv3 loss showed the highest recall and mAP(@0.5) scores and also achieved the second-best score in the F1 composite performance metric, differing by only 0.001 from the optimal value. Figure 10 illustrates the performance of the DIOU, Focal EIOU, and WIOUv3 loss functions on the validation set over each training epoch. From the above experiments, it is clear that the WIOUv3 loss can more accurately detect potential contraband, making it a more suitable loss function for X-ray prohibited item detection applications.

## 4. Conclusions

In response to the common problem of low recall in existing deep learning models for X-ray prohibited item detection tasks, we propose an object detection model called ScanGuard-YOLO based on the YOLOv5 architecture. This model combines the RFB-s, efficient RepGFPN, dynamic head modules, and the WIOUv3 loss function. Experimental results show that the RFB-s module significantly improved precision, while the efficient RepGFPN and dynamic head modules were crucial for improving recall. Optimal results were achieved by combining the WIOUv3 loss function with these three modules. Compared to current state-of-the-art detection models, ScanGuard-YOLO exhibited higher recall and F1 score, showing promising applications in practical X-ray prohibited item detection. It has the potential to assist human operators in accelerating the detection process.

In future work, inspired by the Swin Transformer [33], we plan to improve the backbone network architecture. We aim to improve accuracy while reducing model parameters and computational overhead, thereby increasing detection and recognition speed.

## Figures and Tables

**Table 1:** Detailed Structure of ScanGuard-YOLO. Channel: input and output channels for each block; In: input image size; Out: output image size; Input: input for each block; ©: channel concatenation operation; UP(·): 2x upsampling; F(·): output of the corresponding module; N: serial number of the module.

Backbone							
N	Block	Filter	Stride	Channel	In	Out	Input
1	CBS	6 × 6	2	3/32	(H, W)	(H/2, W/2)	X-ray image
2	CBS	3 × 3	2	32/64	(H/2, W/2)	(H/4, W/4)	F(1)
3	C3	-	-	64/64	(H/4, W/4)	(H/4, W/4)	F(2)
4	CBS	3 × 3	2	64/128	(H/4, W/4)	(H/8, W/8)	F(3)
5	C3	-	-	128/128	(H/8, W/8)	(H/8, W/8)	F(4)
6	CBS	3 × 3	2	128/256	(H/8, W/8)	(H/16, W/16)	F(5)
7	C3	-	-	256/256	(H/16, W/16)	(H/16, W/16)	F(6)
8	CBS	3 × 3	2	256/512	(H/16, W/16)	(H/32, W/32)	F(7)
9	C3	-	-	512/512	(H/32, W/32)	(H/32, W/32)	F(8)
10	RFB-s	-	-	512/512	(H/32, W/32)	(H/32, W/32)	F(9)
**Neck**							
**N**	**Block**	**Filter**	**Stride**	**Channel**	**In**	**Out**	**Input**
11	CBS	1 × 1	1	512/256	(H/32, W/32)	(H/32, W/32)	F(10)
12	CBS	3 × 3	2	256/256	(H/16, W/16)	(H/32, W/32)	F(7)
13	Concat	-	-	512/512	(H/32, W/32)	(H/32, W/32)	F(11©12)
14	CSPStage	-	-	512/256	(H/32, W/32)	(H/32, W/32)	F(13)
15	Upsample	-	-	256/256	(H/32, W/32)	(H/16, W/16)	UP(F(14))
16	CBS	3 × 3	2	128/128	(H/8, W/8)	(H/16, W/16)	F(5)
17	Concat	-	-	640/640	(H/16, W/16)	(H/16, W/16)	F(7©15©16)
18	CSPStage	-	-	640/256	(H/16, W/16)	(H/16, W/16)	F(17)
19	Upsample	-	-	256/256	(H/16, W/16)	(H/8, W/8)	UP(F(18))
20	Concat	-	-	384/384	(H/8, W/8)	(H/8, W/8)	F(5©19)
21	CSPStage	-	-	384/128	(H/8, W/8)	(H/8, W/8)	F(20)
22	CBS	3 × 3	2	128/128	(H/8, W/8)	(H/16, W/16)	F(21)
23	Concat	-	-	384/384	(H/16, W/16)	(H/16, W/16)	F(18©22)
24	CSPStage	-	-	384/256	(H/16, W/16)	(H/16, W/16)	F(23)
25	CBS	3 × 3	2	256/128	(H/16, W/16)	(H/32, W/32)	F(18)
26	CBS	3 × 3	2	256/128	(H/16, W/16)	(H/32, W/32)	F(24)
27	Concat	-	-	512/512	(H/32, W/32)	(H/32, W/32)	F(14©25©26)
28	CSPStage	-	-	512/512	(H/32, W/32)	(H/32, W/32)	F(27)
**Head**							
**N**	**Block**	**Filter**	**Stride**	**Channel**	**In**	**Out**	**Input**
29	CBS	1 × 1	1	128/128	(H/8, W/8)	(H/8, W/8)	F(21)
30	CBS	1 × 1	1	256/128	(H/16, W/16)	(H/16, W/16)	F(24)
31	CBS	1 × 1	1	512/128	(H/32, W/32)	(H/32, W/32)	F(28)
32	Dynamic Head	-	-	256/128	-	(H/8, W/8)	F(29,30)
33	Dynamic Head	-	-	384/128	-	(H/16, W/16)	F(29,30,31)
34	Dynamic Head	-	-	256/128	-	(H/32, W/32)	F(30,31)

**Table 2:** Results of comparison experiments on the OPIXray dataset.

Model	Precision	Recall	mAP (@0.5)	F1	Parameters (M)	GFLOPs
Baseline [15]	0.924	0.861	0.891	0.891	7.0	16.0
YOLOv5m [15]	**0.934**	0.881	0.903	0.907	20.9	48.3
YOLOv6s [25]	0.903	0.876	0.905	0.889	18.5	45.3
YOLOv7tiny [10]	0.887	0.838	0.866	0.862	6.0	13.2
YOLOv7 [10]	0.922	0.858	0.875	0.889	36.5	103.3
YOLOv8s [20]	0.918	0.864	0.902	0.89	11.1	28.7
ScanGuard-YOLO	0.923	**0.906**	**0.914**	**0.914**	13.1	24.1

**Table 3:** Results of comparison experiments on the HiXray dataset.

Model	Precision	Recall	mAP (@0.5)	F1	Parameters (M)	GFLOPs
Baseline [15]	0.861	0.835	0.842	0.848	7.0	16.0
YOLOv5m [15]	**0.88**	0.834	0.852	0.856	20.9	48.3
YOLOv6s [25]	0.85	0.854	0.849	0.852	18.5	45.3
YOLOv7tiny [10]	0.85	0.841	0.837	0.845	6.0	13.2
YOLOv7 [10]	0.875	0.848	**0.858**	**0.861**	36.5	103.3
YOLOv8s [20]	0.851	**0.857**	0.854	0.854	11.1	28.7
ScanGuard-YOLO	0.862	0.855	**0.858**	0.858	13.1	24.1
ScanGuard-YOLOm	0.885	0.851	0.862	0.868	37.8	69.9

**Table 4:** Results of ablation experiments.

A	B	C	D	Precision	Recall	mAP (@0.5)	F1	Parameters (M)	GFLOPs
				0.924	0.861	0.891	0.891	7.0	16.0
✓				0.917	0.875	0.889	0.896	9.2	19.5
✓	✓			0.909	0.869	0.892	0.889	9.2	19.5
✓		✓		0.906	0.881	0.891	0.893	12.3	23.4
✓			✓	**0.93**	0.862	0.895	0.895	10.1	20.2
✓	✓		✓	0.922	0.873	0.897	0.897	10.1	20.2
✓	✓	✓		0.912	0.887	0.906	0.899	12.3	23.4
✓		✓	✓	0.922	0.894	0.897	0.908	13.1	24.1
✓	✓	✓	✓	0.923	**0.906**	**0.914**	**0.914**	13.1	24.1

**Table 5:** Comparative experimental results of different detection heads on the baseline model.

a	b	c	d	Precision	Recall	mAP (@0.5)	F1	Parameters (M)
				0.924	0.861	0.891	0.891	7.0
✓				0.92	0.88	0.897	0.9	13.6
	✓			0.925	0.878	0.898	0.901	11.9
		✓		**0.926**	**0.889**	0.909	**0.907**	11.1
			✓	0.917	0.886	**0.91**	0.901	10.1

**Table 6:** Comparative experimental results using different detection heads on the combined module.

a	b	c	d	Precision	Recall	mAP (@0.5)	F1	Parameters (M)
				0.92	0.873	0.897	0.896	10.0
✓				0.908	0.884	0.899	0.896	16.6
	✓			**0.923**	0.864	0.897	0.893	14.9
		✓		0.92	0.9	0.908	0.91	14.1
			✓	**0.923**	**0.906**	**0.914**	**0.914**	13.1

**Table 7:** Impact of input channel numbers on dynamic head performance.

Channel	mAP (@0.5)	Parameters (M)	GFLOPs
32	0.896	7.3	16.8
64	0.907	7.9	17.8
128	0.91	10.1	19.8
256	0.909	18.8	24.3
512	**0.918**	52.7	35.1

**Table 8:** Ablation study on the number of dynamic head modules.

The Number of Modules	mAP (@0.5)	Parameters (M)	GFLOPs
1	0.902	7.6	16.9
2	0.906	8.1	17.5
3	0.906	8.6	18.1
4	0.903	9.1	18.7
5	0.908	9.6	19.2
6	**0.91**	10.1	19.8
7	0.906	10.6	20.4
8	0.908	11.1	21

**Table 9:** Comparison of experimental results of different loss functions on the baseline model.

Loss Function	Precision	Recall	mAP (@0.5)	F1
DIOU [31]	**0.924**	0.873	0.892	0.898
CIOU [31]	**0.924**	0.861	0.891	0.891
EIOU [29]	0.918	0.87	0.894	0.893
SIOU [13]	0.921	0.869	**0.895**	0.894
Focal DIOU [29,31]	0.904	0.861	0.893	0.882
Focal CIOU [29,31]	0.908	**0.885**	**0.895**	0.896
Focal EIOU [29]	0.918	0.869	**0.895**	0.893
Alpha DIOU [32]	0.908	0.854	0.873	0.88
Alpha CIOU [32]	0.908	0.843	0.873	0.874
Alpha EIOU [29,32]	0.895	0.858	0.881	0.876
Alpha SIOU [13,32]	0.889	0.835	0.869	0.861
WIOUv3 [19]	0.918	0.884	0.891	**0.901**

**Table 10:** Comparative experimental results with different loss functions on combined modules.

Loss Function	Precision	Recall	mAP (@0.5)	F1
DIOU [31]	0.938	0.893	0.912	**0.915**
CIOU [31]	0.923	0.889	0.908	0.906
EIOU [29]	0.905	0.889	0.91	0.897
SIOU [13]	0.928	0.891	0.91	0.909
Focal CIOU [29,31]	0.903	0.891	0.909	0.897
Focal EIOU [29]	**0.94**	0.874	0.912	0.906
WIOUv3 [19]	0.923	**0.906**	**0.914**	0.914

## Data Availability

All data are available from the corresponding author upon request.
